# Middle-aged individuals with thalidomide embryopathy have undergone few surgical limb procedures and demonstrate a high degree of physical independence

**DOI:** 10.1371/journal.pone.0186388

**Published:** 2017-10-20

**Authors:** Shadi A. Ghassemi Jahani, Aina Danielsson, Jon Karlsson, Helena Brisby

**Affiliations:** 1 Department of Orthopaedics, Kungälv Hospital, Kungälv, Sweden; 2 Department of Orthopaedics, Institute of Clinical Sciences, Sahlgrenska Academy, University of Gothenburg, Gothenburg, Sweden; 3 Department of Orthopaedics, Sahlgrenska University Hospital, Gothenburg, Sweden; IRCCS E. Medea, ITALY

## Abstract

**Background:**

Thalidomide is known to have induced thalidomide embryopathy (TE) in more than 10,000 live-born children worldwide between 1957–1962.

**Aim:**

The aim of this study was to investigate the need for orthopaedic surgery and limb orthosis in relation to function and physical independence in middle-aged individuals with TE.

**Methods:**

13 women/18 men with a mean age of 45.8 (SD 1.1) years were included. Information about limb surgery, the use of orthotic devices, jobs, accommodation, disability adjustments and personal assistants was collected. Physical function was measured by a modified general function score. The time needed for activities of daily living (ADL) was collected. Individuals with proximal focal femoral deficiency, PFFD, and participants in need of home or work adaptations were compared with the rest of the group.

**Result:**

31 surgical procedures had been performed in the extremities. Three individuals were in need of personal assistance and seven had disability-adjusted homes. 28 individuals were working and 24 reported participation in exercises. Those with PFFD had significantly lower function score and needed a significantly longer time for ADL in the morning (p = 0.001 and p = 0.032). The group in need of home or work adjustments had significantly lower function score and needed longer time for morning ADL (p = 0.012 and p = 0.009).

**Discussion:**

Few orthopaedic procedures had been performed. The TE individuals except the ones with PFFD and those in the need of disability adjustments, were mostly active workers, reported good physical function and participated in exercises, despite limb malformations.

## Introduction

The incidence of congenital limb defects, also known as limb reduction defects, is low and stable, approximately six per 10,000 live births in Canada and four per 10,000 in Sweden [1, 2]. Congenital defects, especially those of the limbs, usually develop in the early prenatal period [3, 4]. The exact cause is not always identified; however, unspecific gene translocations, amniotic bands during the later trimester and smoking have been identified as possible causes [5–7]. It has been documented that congenital abnormalities caused by teratogenic drugs account for less than 1% of the total congenital abnormalities [8]. One of the most well-known and potent teratogenic drugs causing severe limb defects in humans is thalidomide.

Thalidomide was used as a sedative drug for pregnant women at the end of the 1950s and the beginning of the 1960s in Sweden [9] as well as in other countries [10]. When ingested during early pregnancy, the drug increases both the rate of extremity malformations and defects in other organs [9, 11, 12]. The drug is still in use, but it is currently used for conditions such as multiple myeloma, complications of leprosy and Crohn’s disease, due to the inhibitory effects of TNF-a [10].

Malformations of the extremities often lead to disabilities that may affect occupational and educational choices [13] and may increase contact with the health-care system.

The aim of this study was to investigate patient-reported need for orthopaedic care, such as surgical corrections of the limbs and the use of orthoses, the need for walking aids and disability adjustment of the workplace and home, as well as activities of daily living, ADL, and physical function, in a group of middle-aged patients with thalidomide embryopathy (TE).

## Material and methods

### Patients

Thirty-one individuals, 13 women and 18 men with a mean age of 45.8 (SD 1.1), all of whom responded to a mailed request via a TE patient organisation (the Thalidomide Society in Sweden) [9], were included in the study.

Individuals with more pronounced limb deficiency, particularly in the lower extremities, verified by computed tomography (CT), proximal focal femoral deficiency (PFFD, n = 5), [9], were compared with the rest of the group (n = 26).

PFFD comprises a deficiency of the femur and sometimes also of the hip joint, which, depending of the extent of the malformation, often results in a shortening of the leg. In the most extreme cases there is neither a thigh nor a hip joint, causing the lower leg to begin in the groin level and leaving the foot at a normal knee level.

In this study group, four of the five patients with PPFD had bilateral significant shortening of the femur as well as bilateral lower limb malformations which further affected their limb length. The fifth patient had a unilateral PFFD with a moderate shortening of the femur, and on the other side a femoral head necrosis of unknown reason, i.e. a unilateral limb shortening.

Individuals who had disability-adapted living and/or work and/or a personal assistant (Group A) were compared with individuals without these adjustments and arrangements in their daily life (Group B).

This study was approved by the local human research ethics committee at the University of Gothenburg: Ö 556–03. All participants received written and verbal information about the study and gave their written informed consent.

### Questionnaires

Information on previous and current diseases, all types of surgery for the extremities, the need for walking aids or wheelchair transportation indoors and outdoors, the use of external orthoses, personal assistance (defined as a person employed to provide assistance) and disability adjustments in the home or workplace was collected using a patient-administered questionnaire. The participants also filled in a questionnaire about the time needed for morning preparations, including dressing and morning toilet, i.e. activity of daily life (ADL), and, similarly, for the evening procedures.

The general function level in terms of different activities of daily life was collected using a modified “general function score” (GFS) questionnaire [14]. The General Function Score was designed as a disease-specific questionnaire, used for the evaluation of function in patients with low back pain (LBP), and it originally contained 17 questions. In the present study, a “Modified General Function Score” was used. The specific question in terms of LBP was excluded, as the focus was function in general and not specifically LBP. The alternatives for each question, after modification were: “can perform”, “can perform with difficulty”, “cannot perform” or “need help of an assistant”. The answers are valued between “0” for “can perform” and “3” for “need help of an assistant” and the composite sum for all questions is presented as the percentage with 0 representing maximum function, i.e. highly independent, and 100 representing minimal function, i.e. highly dependent.

Questions on the physical load at the workplace and participation in and the degree of physical activity during leisure time were retrieved according to the WHO questionnaire [15]. These two questions are based on the level of strain during working hours and during leisure time. The answers related to load at work are expressed as “sedentary”, “light work with some physical activity”, “relatively heavy work” and “heavy manual work” and, regarding the degree of physical activities during leisure time, as “sedentary”, “light exercises and training”, “regular training” and “serious training and competitive sports” respectively.

All the questionnaires were completed on a single day for each patient during an outpatient visit to the Sahlgrenska University Hospital, Gothenburg, Sweden.

### Statistics

All the data have been processed in SPSS version 23. For comparisons between the groups, the Mann-Whitney U test has been used for continuous variables and Fisher’s exact test for categorical variables. All tests were two-tailed and conducted at the five per cent significance level.

## Results

### Other diseases/disorders

Fifteen individuals reported other diseases such as hypertension, sleep apnoea syndrome, migraine, diabetes, gastrointestinal reflux, breast cancer, epilepsy, fibromyalgia and stroke.

None of these conditions affected and/or added to the need for orthopaedic care in any of the individuals.

### Surgical procedures

#### Upper extremities

Twenty-three procedures on the upper limbs had been performed on 18 (58%) individuals and ten of these individuals had undergone the surgery (-ies) before the age of 18 years.

The most frequent procedure was thumb surgery; nine surgical procedures on the thumbs had been performed in seven patients. Three procedures were amputations and the remaining six were reconstructive thumb surgery. Decompression of the median nerve at wrist level due to carpal tunnel syndrome (CTS) was performed in four individuals and bilaterally in two of them, making a total of six procedures (Table 1).

**Table 1:** Different surgical procedures on the limbs after birth in 31 individuals with thalidomide embryopathy.

Limb surgery performed	Procedures	N. pts (%)/N. op (%)
Upper limbs	Acromyoplasty	1(3)/1(3)
	Arm surgery (unspecified)	1(3)/1(3)
	Decompression of medial nerve (for two pts bilaterally)	4(13)/ 6(19)
	Transposition of nerves	1(3)/1(3)
	Wrist surgery	2(6)/ 3(10)
	Other thumb procedures	5(16)/ 6(19)
	Amputation of thumb	2(6)/3(10)
	Surgery on the fingers	2(6)/2(6)
**Lower limbs**	Hip joint replacement	1(3)/1(3)
	Hip reconstruction surgery during childhood	1(3)/1(3)
	Knee arthrodesis	1(3)/1(3)
	Foot ligament reconstruction	1(3)/1(3)
	Foot amputation	1(3)/1(3)
	Leg lengthening	1(3)/1(3)

Age at surgery for upper limbs: 10 <18 years, 7 >18 years, One unknown

Age at surgery for lower limbs: 4 >18 years, 5<18 years

#### Lower extremities

Eight lower limb surgeries had been performed in eight of 31 individuals (24%) and five of these individuals were younger than 18 years at the time of surgery. The majority (6/8) of lower extremity procedures were performed within the group of the five individuals with PFFD. All the procedures performed on the group with PFFD, for both lower and upper extremities, apart from one CTS surgery, had been performed before the age of 18 years, Table 2.

**Table 2:** Surgeries performed on the five persons with proximal focal femoral deficiency (PFFD).

Extremities	Type of surgery	Age at the time of surgery
	One CTS* in one patient	>18
**Upper limbs**	Three surgeries on the wrist in two patients	<18
	One unspecific arm surgery in one pt	<18
	Amputation of a foot in one patient	<18
**Lower limbs**	Arthrodesis of a knee in one patient	<18
	Two surgeries for a clubfoot in two patients	<18
	Hip surgery during childhood in two patients	<18

* CTS: Carpal Tunnel Syndrome

### Orthopaedic devices and aids

#### Walking aids and orthotic devices

Walking aids were used by 11 of 31 individuals (32%) on some occasions every day, but only five (13%) needed some type of walking aid indoors.

Twenty-seven (87%) of the individuals did not use any orthotic devices such as an orthosis or prosthesis for their lower limbs. No one used any orthotic devices for the upper extremities. Two individuals with PFFD were wheelchair bound and one individual occasionally used a wheelchair. Three patients used a lower leg prosthesis on one leg on one side. For details, see Table 3.

**Table 3:** The need for different types of aid, disability adjustments and assistance to manage locomotion and daily activities in the total of 31 individuals with TE.

	TE, n = 31, n(%)	No-PFFD, n = 26, n(%)	PFFD, n = 5, n(%)
**Walking aid**			
Indoors*	5(13)	0	5(100)
Outdoors*	6(19)	1(4)	3(60)
**Orthotic devices**			
Lower limb	4(13)	1(4)	3(60)
**Disability adjustment home**			
yes	7(23)	3(12)	4(80)
Would like	1(3)	0	1(20)
Disability adjusted workplace**			
yes	9(31)	6(23)	3(60)
**Personal assisstant**			
yes	3(10)	2(8)	1(20)

Thalidomide embryopathy (TE) (n = 31) individuals with and without proximal focal femoral deficiency (PFFD) (n = 5) and (n = 26) respectively

* Two wheelchair-bound individuals

** 29 answered the question

### Personal assistance, disability-adjusted home and workplace

Three individuals (10%) had help from a personal assistant for four, five and 16 hours a day respectively. Disability adjustments to their homes had been made for seven of the total of 31 individuals (23%). Four of these seven were individuals with PFFD (Table 3).

A total of twelve individuals who have had home and/or workplace adaptations due to disability and/or support in the form of personal assistance formed Group A (n = 12). These individuals were compared with the individuals without these adjustments in their daily lives, Group B (n = 19).

### Activity of daily living (ADL) and modified general function scores (GFS)

**Fig 1:**
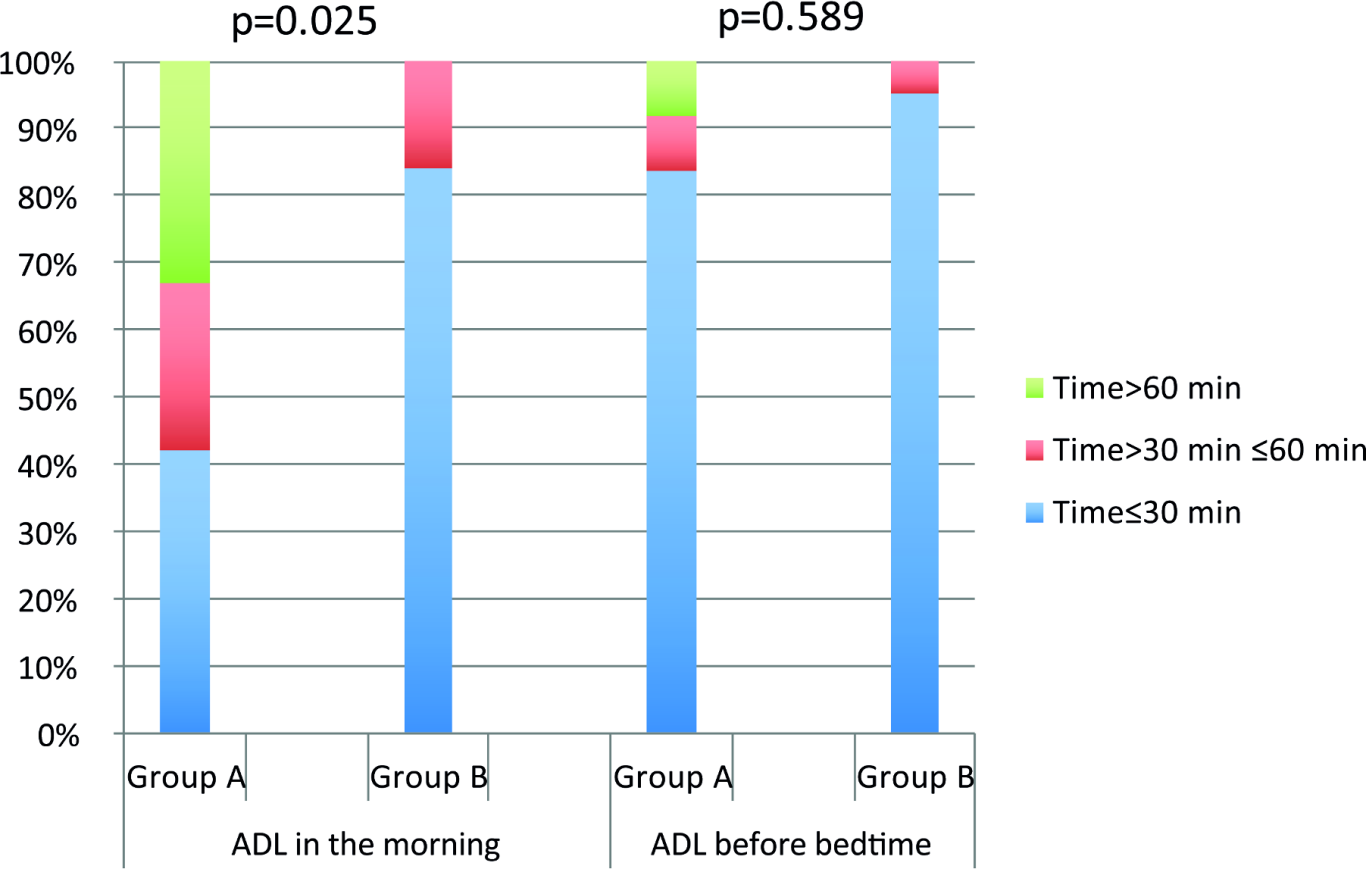
Time needed for ADL for Group A (TE individuals with a disability-adjusted home and/or workplace and/or a personal assistant), (n = 12) and Group B (no need for disability adjustment and/or personal assistant) (n = 19). Significantly more individuals in Group A stated that they needed longer for ADL in the morning than those in Group B (p = 0.009).

The time needed for ADL for the whole group revealed that almost 70% of the TE individuals did not need more than 30 minutes to prepare in the morning and 90% managed ADL before bedtime in 30 minutes or less (Fig 1).

Eighty per cent of individuals with severe lower limb malformation (PFFD) needed more than 30 minutes for morning preparation, compared with 23% of those without, p = 0.0032 (Table 4).

**Table 4:** Physical function measured by modified GFS and ADL for the total study group individuals with PFFD and without PFFD. Comparison calculated between individuals with PFFD and without PFFD. mean (SD)/range, n(%).

Physical function	All, n = 31	No PFFD, n = 26	PFFD, n = 5	p-values
**Modified GFS**	12.6(21)/(0–66)	7.2(17.5)/(0–66)	41(15)/(25–56)	0.001
**Time for ADL**				
**Morning**				
≤ 30 minutes	21(68)	20(77)	1(20)	
> 30 min ≤ 60 minutes	6(19)	4(15)	2(40)	0.032
> 60 minutes	4(13)	2(8)	2(40)	
**Evening**				
≤ 30 minutes	28(90)	24(92)	4(80)	
> 30 min ≤ 60 minutes	2(7)	1(4)	1(20)	0.422
> 60 minutes	1(3)	1(4)	0	

A longer time (more than 30 minutes) for morning ADL was also taken by individuals in Group A, for whom additional help was needed, compared with the rest, Group B, 58% and 16% respectively, p = 0.009. However, there was no statistically significant difference between Group A and B or between the groups with PPFD vs. non-PFFD in terms of ADL before bedtime.

The modified GFS for the total group of TE individuals was a mean of 12.6 (range 0–66), (Table 4). The mean score for the modified GFS was significantly lower for the group without PFFD, 7 (range 0–66), compared with those with PFFD, 41 (range 25–56)), (p = 0.001). The GFS for Group A was significantly higher compared with the rest of the group (Group B), mean 28 (range 0–65) compared with 3 (range 0–22) (p = 0.012).

### WHO questionnaire

Almost half the individuals mainly performed sedentary work, 13/28 (46%), while 7/28 (25%) classified their present work as relatively heavy or heavy. During leisure time, 24/31 (77%) reported participation in some type of physical exercise/training. The detailed result of strain level at work and activity level during leisure time according to the WHO questionnaire is presented in Table 5.

**Table 5:** Comparison between the total TE group (n = 31), individuals with PFFD and without PFFD regarding physical activity level at work and in spare time according to WHO questionnaire n(%).

Working time	All	Not PFFD	PFFD
Sedentary	13(46)	10(42)	3(75)
Light work with some physical activity	8(29)	7(29)	1(25)
Relatively heavy work	5(18)	8(21)	0
Heavy manual work	2(7)	2(8)	0
**Leisure time**			
Mainly sedentary	7(23)	4(15)	3(60)
Light exercise and training	14(45)	13(50)	1(20)
Regular training and exercise	9(29)	8(31)	1(20)
Serious training and competitive sports	1(3)	1(4)	0

## Discussion

The present study demonstrates that the studied group of individuals with thalidomide embryopathy (TE) had undergone relatively few surgical procedures on their limbs until middle age and only about a third needed disability adjustments in their home and/or workplace. Moreover, the study group reported a high level of employment and most of the individuals were actively involved in some type of physical activity in their leisure time. More severe limb malformations, however, reduced the individual’s independence and increased the time taken to perform common ADL procedures.

The number of limb surgeries due to malformations was generally low and more so after the age of 18 years. The thalidomide embryopathy occurred at the beginning of the 1960s, i.e. before the modern era of limb reconstruction including lengthening procedures. None of these individuals had undergone surgical procedures that are currently performed and this is one reason for the relatively small number of surgeries and for remaining limb length inequality with the need for adaptation of mobility and daily life. The finding that surgical procedures on the upper extremities were more common than lower limb surgeries may be due to the higher prevalence of limb deficiencies and other congenital malformations of the upper limbs [4, 5, 9]. Further, surgeries of the upper extremities later in life, related to overuse or a modified movement pattern of arms/hands, probably also added to this number [16]. In TE, the thumbs have various malformations; while some TE have three phalangeal thumbs, other TE might have no thumb at all but five or fewer parallel fingers, without any opportunity for thumb opposition movements [9]. Nine surgeries were performed on the thumbs in seven individuals, most probably to reconstruct and create a functional thumb with opposition.

Carpal tunnel release was a relatively frequent procedure in this cohort and all these procedures were performed after the age of 18 years. In a recent report from Japan, a high frequency of carpal tunnel syndrome (CTS) in individuals with TE has been noted [16]. The absence, hypoplasia or dysplasia of the radius is a common malformation in the upper extremities of TE individuals [4] and a subsequent wrist deformity might cause compression of the median nerve at wrist level. Whether CTS is caused by the limb malformations and the resulting overuse or changed use of the wrists, or is a consequence of the embryonic damage caused by the drug or a combination of these is however unclear.

Unlike the upper limb surgery, most of the lower limb surgeries were performed during childhood or adolescence. The low rate of limb surgeries after the age of 18 can probably be explained by the fact that most surgeries to the lower limbs were reconstruction procedures and were therefore performed at a young age.

Approximately 13% of the TE individuals used some type of orthotic device and they were all used for the lower limbs. No one in this study cohort was using orthotic devices for the upper limbs, even though four individuals totally lacked, or had very short upper extremities on both sides or one side [9]. This may be explained by the fact that the available orthoses have not been as good as using the existing lower or upper limbs for gripping and/or finding other ways to manage. The group with TE have often developed modified ways to use their malformed extremities or to use their mouths or teeth instead as gripping tools [17].

Considering the different malformations in the upper extremities and the need for additional help in life in almost 40% of the participants, it was somewhat unexpected to find that most of the individuals in this cohort needed less than 30 minutes for their morning and evening ADL procedures. The individuals in need of personal assistance and/or a disability-adjusted home and/or workplace, however, reported a somewhat longer time to perform their personal morning hygiene and get ready for daily activities, i.e. ADL morning procedures.

The same relationship was found for the group with PFFD, indicating that more severe malformations require extra time in order to manage ADL procedures. However, independence did not appear to be purely related to the degree of limb malformations, as only one individual with PFFD reported the need for a personal assistant.

A modified general function score was used to measure physical disability and the result for this score varied between 0–66 in the investigated group, indicating that the individuals rated their function between totally independent (0%) and relatively dependent (66%). As this score has not previously been used for this type of patient group, it is not possible to compare it with previous findings. However, a range between 0–66 on the modified GFS still indicates relatively high self-rated independence in terms of physical function within this group.

In the five individuals with PFFD, the modified general function score indicated somewhat more dependence in terms of physical activities. The range was, however, still between 25–56%, indicating that most tasks could be carried out by the individual without any assistance.

When looking at strain at work and during leisure, the results revealed a high rate of employment and a high level of participation and performance in regular training and exercise within the group.

The main limitation of this study was the small number of participants. All identified living adults with TE nationally that accepted to participate were included, but a higher response rate would of course have been desired. However, a cohort of 31 middle-aged individuals with TE can be regarded as a highly unique cohort and, compared with previous studies in individuals with this condition, this is a relatively large study group. The fact that this cohort consisted of the responders to a request via a patient association may have caused a patient selection bias, with a possibility that the responding individuals had better function than the overall group of TE individuals. This is, however, only a suspicion, as permission for us to retrieve information about the non-responding group was not granted.

All the information about surgeries and orthoses is self-reported by the patients. The true nature of previous surgeries could not be captured, as it was not possible to retrieve the old charts and/or radiographs. Furthermore, the physical function and activity level could possibly have been affected by other diseases, but none of the reported diseases were directly linked to any of the orthopaedic or surgical care given.

In conclusion, few orthopaedic surgeries had been performed in this group of middle-aged patients with TE. The majority were in employment and had a relatively good physical activity level during their leisure time. Most of these individuals regard themselves as being mainly independent in terms of physical function, despite some level of disability and impairment due to limb malformations.
